# Anti-tumor effect of Huaier extract against neuroblastoma cells *in vitro*

**DOI:** 10.7150/ijms.48219

**Published:** 2021-01-01

**Authors:** Dong-qing Xu, Xiao-jun Yuan, Hidemi Toyoda, Masahiro Hirayama

**Affiliations:** 1 Department of Pediatric Hematology/Oncology, Xin Hua Hospital Affiliated to Shanghai Jiao Tong University School of Medicine, Shanghai 200092, China; 2 Department of Pediatrics, Mie University Graduate School of Medicine, 2-174, Edobashi, Tsu, Mie, Japan

**Keywords:** Neuroblastoma, MEK/ERK, mTOR, Huaier Extract, Apoptosis

## Abstract

Huaier extract, the main active constituent proteoglycan, has anti-tumor activity in various experimental and clinical settings. However, the potential anti-neuroblastoma and associated mechanisms have not been investigated. Therefore, in this study, we aimed to elucidate the potential role of Huaier extract in 3 human neuroblastoma cell lines. Our study demonstrated that incubation with Huaier extract resulted in a marked decrease in cell viability in a dose-dependent manner. Huaier extract induced cell cycle arrest at G0/G1 phase in neuroblastoma and decreased the cell cycle related protein expression of cyclin D3. Western blotting analysis also showed that Huaier extract induced neuroblastoma cell apoptosis and autophagy. Signaling analysis indicated that Huaier extract suppressed the MEK/ERK and mTOR signaling pathways simultaneously. In conclusion, we verify that Huaier extract causes cell proliferation inhibition, apoptosis, autophagy, and cell cycle arrest in G0/G1 phase via MEK/ERK and mTOR signaling. Huaier extract may act as a complementary agent for treating neuroblastoma.

## Introduction

Neuroblastoma (NB), an aggressive neural crest derived malignancy, is the most common pediatric solid tumor with the majority of cases presenting by 5 years of age, accounting for approximately 15% deaths of total pediatric cancers 1. NB exerts a wide range of clinical behaviors from spontaneous regressions especially in children less than 1 year to lethal outcome even with intense multimodal therapy due to biologic heterogeneity 2,3. Current multimodal treatment options for NB involves induction chemotherapy, surgical resection of the primary tumor, radiation, high-dose myeloablative chemotherapy with autologous stem-cell transplantation (SCT), immunotherapy using anti-GD2 antibody and differentiation therapy using 13- cis retinoic acid (CRA) for targeting the residual tumor 4. With advanced multimodal treatment approaches, prognosis of NB has improved evidently in the last two decades, however, a significant number of high-risk children suffered a depressing long-term survival rate due to recurrence or development of resistance to conventional therapy 5. Therefore, it remains necessary to explore alternative treatment strategies especially for those who are diagnosed with high-risk NB.

Considering the implicated molecular pathogenesis, targeted therapies using inhibitors of molecules has been a major advance in development NB treatment except for the conventional treatment choices. Generally, overactivation of signaling pathways contribute to carcinogenic mechanism in many malignant diseases. NB cells, like many cancer cells, possess an aberrant activation mammalian target of rapamycin (mTOR) signaling pathway which has been evidenced in our previous studies 6. mTOR forms one of two different complexes, mTOR complex 1 (mTORC1) or mTOR complex 2 (mTORC2), which are distinguished by their partner proteins, substrate specificities and sensitivity to rapamycin 7. Dual mTORC1and mTORC2 inhibitor, AZD8055, showed promising anti-tumor activities against NB in our previous study. Preclinical studies were also supportive of the use of mTOR antagonists alongside signal transduction inhibitors or chemotherapy in NB 8. Despite advances in the development of targeted therapies and initial encouraging results, many patients later develop resistance to the targeted drug and relapse due to the activation of alternate pathways. Our previous study also found that NB cells tends to acquire resistance with prolonged application of AZD8055 due to MEK/ERK signaling overactivation which could be overcome by ERK molecular inhibitor (U0126) successfully 9. Therefore, development of agents targeting multiple pathways implicated in the pathogenesis of NB and has lower toxicity profiles are urgently needed.

In recent years, many traditional Chinese herbal medicines (TCM) have exerted promising anti-cancer efficacy and applied in clinical settings as complementary anti-tumor agents 10. Huaier, also known as Trametes robiniophila Murr, has been applied as a TCM for more than 1,600 years. Huaier is isolated from the extract of officinal fungi and the major effective ingredient is identified as proteoglycan, which includes 41.53% polysaccharides, 12.93% amino acids and 8.72% water 11. Numerous evidences suggested that Huaier extract provides potential anti-neoplastic benefits in many types of tumors in experimental or clinical settings 12-15. In lung cancer, Huaier extract inhibited the proliferation and metastasis of cancer cells through down-regulation of MTDH, JAK2/STAT3 and MAPK signaling pathways 12. In cholangiocarcinoma, 5-FU combined with Huaier extract exhibited a synergistic anti-tumor effect via activation and translocation of STAT3 13. In breast cancer, patients orally administrating Huaier granule got longer DFS and showed lower incidence of emotional symptoms 14. In hepatocellular carcinoma, patients who received Huaier granule showed improving recurrence-free survival and decreasing extrahepatic recurrence after radical surgical resection 15. All these evidences demonstrated that Huaier extract may be a promising alternative agent in treatment of cancers alone or combined with other drugs.

The underlying anti-tumor activity and molecular mechanisms of Huaier extract are not yet fully understood, and no studies have investigated the potential role of Huaier extract in human NB cells. In this study, we found that Huaier extract can effectively induce cell-growth inhibition, cell cycle arrest, apoptosis, and autophagy through suppressing the activation of MEK/ERK and mTOR signaling simultaneously in human NB cells, providing new ideas for comprehensive treatment for NB.

## Materials and methods

### Preparation of Huaier extract*.*

The electuary Huaier ointment was provided by Qidong Gaitianli Medicine Co., Ltd. (Qidong, Jiangsu, China). In total, 2 g of the electuary ointment was dissolved in 20 ml complete medium and sterilized with 0.22μm filter to obtain a 100 mg/ml stock solution, which was subsequently stored at ‑20˚C.

### Cell lines and cell culture

3 human NB cell lines, described previously 6, were used in the study. The human NB cell lines were cultured in RPMI1640 (R8758, Sigma) medium supplemented with 10% fetal bovine serum (FBS) (GIBCO). Cells were incubated in a humidified atmosphere at37 °C with 5% CO2.

### Antibodies and reagents

The following antibodies and reagents were used in this study: phospho-mTOR (Ser2448) (#5536, Cell Signaling), phospho-mTOR(Ser2481) (#2974, Cell Signaling), phospho-AKT (Ser473) (#4058, Cell Signaling), phospho-4E-BP1 (Ser65) (#34443, Cell Signaling), anti-MEK (#9122, Cell Signaling), anti-ERK1/2 (#9102,Cell signaling), phospho-MEK (#9121, Cell Signaling), phospho-ERK1/2 (#9106, Cell Signaling), anti-cleaved PARP (#5625, Cell Signaling), anti-cleaved caspase3 (#9602, Cell Signaling), autophagy sampler kit (#4445, Cell Signaling), and cell cycle regulation kit (#9932T Cell Signaling).

### Cell morphology

The NB cells were incubated with Huaier extract at a concentration of 0.2 or 1 mg/ml for 72 h. In order to identify the morphological changes, cells were observed under an Olympus light microscope (CX31‑72C02; Olympus Corporation, Tokyo, Japan), and photomicrographs were captured using an Olympus digital camera (DP72; Olympus Corporation).

### MTT assay

MTT cell counting reagent was obtained from Sigma Aldrich. Cells (5 × 10^3^) were seeded in 100 μl medium in 96 wells plates and pre-incubated for 6 h before the addition of inhibitors. MTT (20 μl, 5 mg/ml) was added into each well. After 3.5 h incubation in a humidified atmosphere at 37 °C with 5 % CO_2_, the culture media was removed, and DMSO (150 μl) was added. The plates were shaken vigorously for 15 minutes and the absorbance at 590 nm was measured using multi-spectrophotometer (Viento, Dainippon, Japan). The optical density was used to determine the cell number from a standard curve. Standard curves were plotted for each cell line for each media. The results are expressed as mean ± SD from 3 independent experiments.

### Western blotting

Cytoplasmic extracts were obtained as previously reported 6. The proteins (20 μg/lane) were run on 7.5, 10 or 15 % sodium dodecyl sulfate-polyacrylamide gel electrophoresis followed by semi-dry transfer to PVDF membrane (Invitrogen, Carlsbad, CA). Transferred PVDF blots were pretreated with 5 % non-fat dry milk in TBST containing 0.1 % Tween-20 and incubated with primary antibody (1:1000-3000) at 4 °C overnight. The membrane was then washed 3 times with TBST and incubated with horseradish peroxidase-conjugated secondary antibody (1:1000-3000) for 1 h at room temperature. For phosphorylated protein, transferred PVDF blots were pretreated with PVDF Blocking Reagent (TOYOBO, Osaka, Japan) for 1 h, and incubated with primary and then with secondary antibody (1:3000-6000) which were diluted with Can Get Signal® Immunoreaction Enhancer Solution (TOYOBO, Osaka, Japan) at room temperature for 1 h. After washing three times again, antibodies bound to protein blots were detected by using Western Lightening Chemiluminescence Reagent Plus (Perkin Elmer Life Science, Boston, MA, USA), visualized on LAS-3000 mini (FUJIFILM).

### Cell cycle analysis

Cell cycle analysis was performed after treatment with/ without Huaier extract for 24 h. Cells (2 × 10^6^) were harvested and fixed in 99.5 % ethanol over night at -20 °C, followed by incubation with 500μl propidium iodide (PI) Triton X-100 solution containing RNase A at room temperature for 30 min in darkness, then the DNA content was analyzed immediately with FACS-can flow cytometry, analyzed by using ModFitLT software.

### Statistical analysis

Statistical analysis was performed using SPSS (IBM Corporation). Statistical significance of differences between groups was evaluated using Student's t-test and two-way ANOVA. A p-value < 0.05 was statistically significant.

## Results

### Huaier extract significantly reduced cell viability of neuroblastoma cells

**Fig 1:**
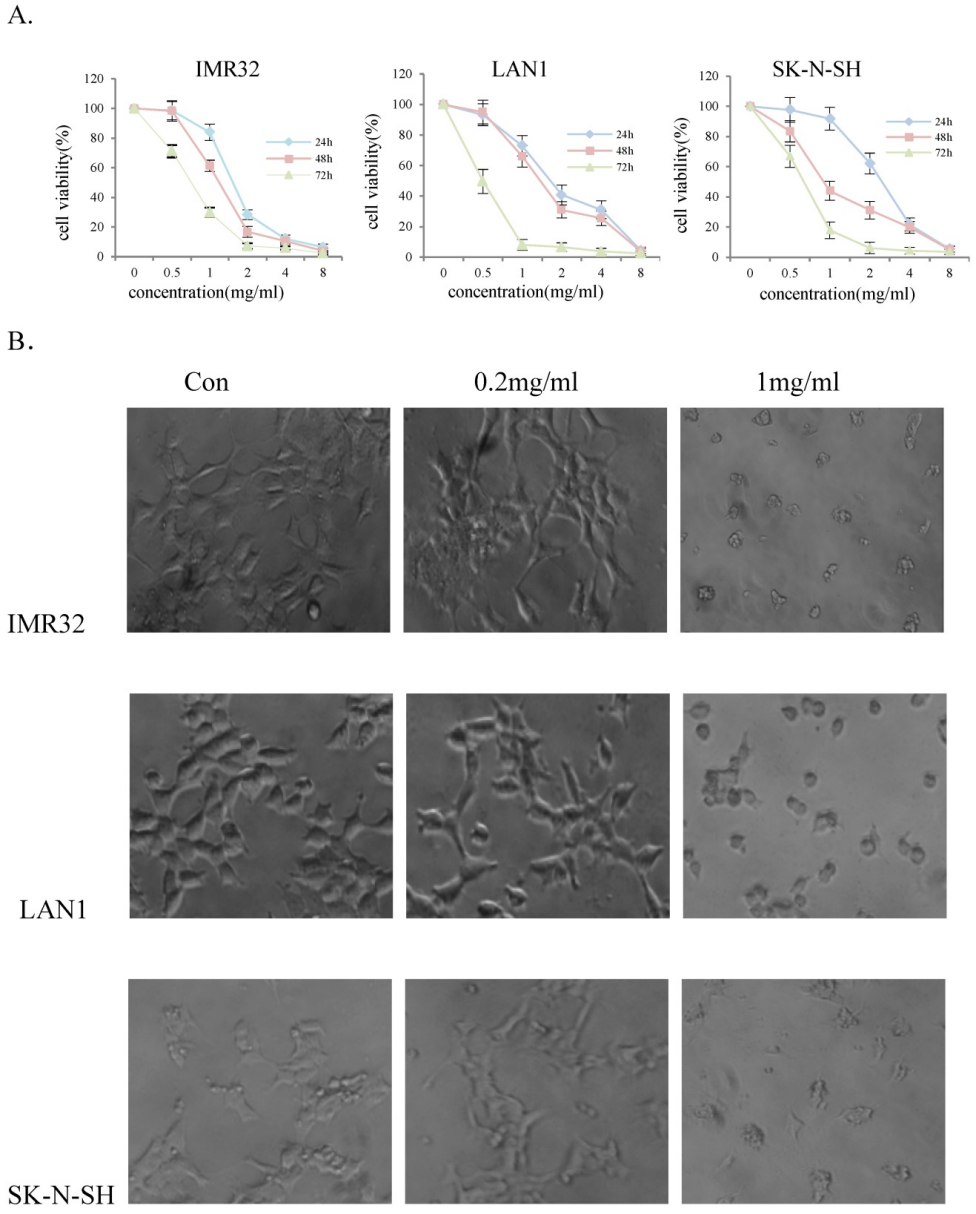
** Huaier extract significantly reduced cell viability of neuroblastoma cells.** The effect of Huaier extract on cell viability was measured by MTT assay. (A) 3 neuroblastoma cell lines (LAN1, SK-N-SH and IMR32) were treated with Huaier extract for 24, 48 and 72 h. Huaier extract significantly inhibited cell viability of the 3 neuroblastoma cell lines in a dose- and time-dependent manner. The experiments were performed in triplicate and data presented as the mean ± SD of three separate experiments. (B) Phase‑contrast images revealing the morphologies of 3 neuroblastoma cells prior and subsequently to treatment with 0.2 mg/ml and 1mg/ml Huaier for 48 h. Magnification 200×.

To evaluate the effect of Huaier extract on NB cell-growth, three NB cell lines, IMR32, LAN1 and SK-N-SH, were incubated in the presence of Huaier extract at 0.5 - 4 mg/ml for 24, 48 and 72 h. As shown in Fig. 1A, Huaier extract significantly decrease cell viability of the 3 NB cell lines in a dose-dependent and time-dependent manner. The viability of IMR32, LAN1 and SK-N-SH cell lines after 2 mg/mL exposure for 72 h decreased by almost 97%, 94% and 96% respectively. As shown in Fig. 1B. Huaier extract caused significant morphological alterations in the human NB cell lines after treatment with Huaier for 48 h with different concentrations. Compared with the untreated cells maintaining a regular shape and size, the majority of the Huaier extract-treated NB cells became irregular-shaped, weaker cell-adhesion and had a vacuolized change of cytoplasm. These morphology changes demonstrated cell damage after Huaier extract treatment.

### Huaier extract induced G0/G1-phase arrest in neuroblastoma cells

**Fig 2:**
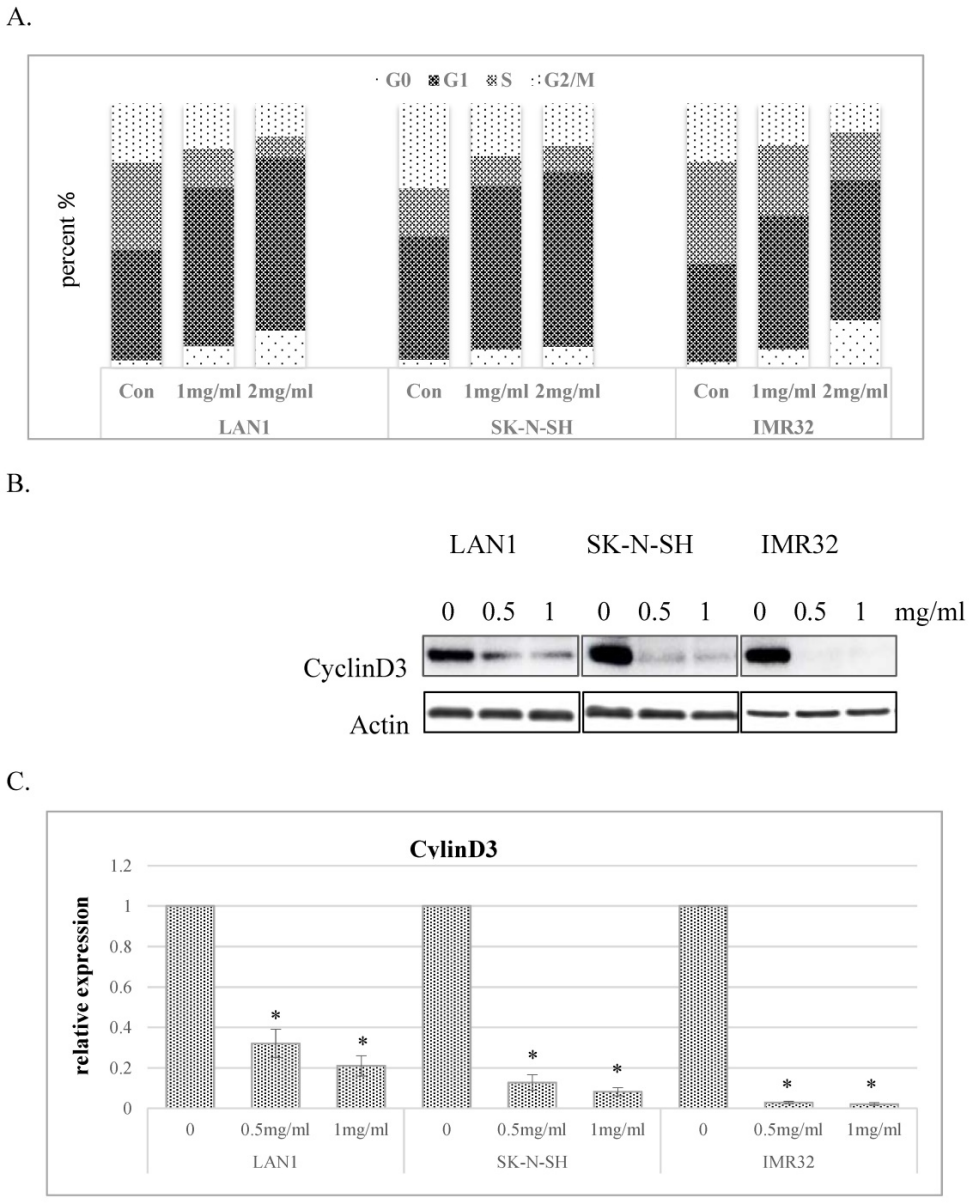
** Huaier extract induced G0/G1 phase accumulation of cell cycle in neuroblastoma cells. A**. The effect of Huaier extract on cell cycle phase distribution in 3 neuroblastoma cell lines treated with/without Huaier extract (0, 1, 2 mg/ml) in RPMI1640 + 10 % FBS for 24 h followed by analysis of cell cycle, as introduced in methods and materials. Cells were stained with PI for 30 min followed by FACS-can flow cytometer. **B**. 3 neuroblastoma cells were incubated in RPMI1640 + 10 % FBS with/without Huaier extract (0, 0.5, 1 mg/ml) for 24h. CyclinD3 were detected by western blotting, so was Actin. **C**. The intensity of CyclinD3 was quantified by ImageJ. After normalization to actin, the relative expression of CyclinD3 versus control group was compared. Data represent mean ± SD. * represents P<0.05.

To explore the mechanisms responsible for the inhibitory effect of cell growth, we investigated the impact of Huaier extract on cell cycle distribution in the human NB cell lines. As shown in Fig. 2A, Huaier extract induced G0/G1 phase accumulation in dose-dependent manner in NB cell lines. Since progression from G1 to S phase in mammalian cells is regulated by D-type cyclins, proteins related to cell cycle regulation were examined by western blotting. As shown in Fig. 2B and 2C, cyclinD3 was downregulated in all 3 NB cell lines treated with Huaier extract, consistent with the accumulation of cells in G0/G1 phase.

### Huaier extract induced apoptosis and autophagy in neuroblastoma cells

**Fig 3:**
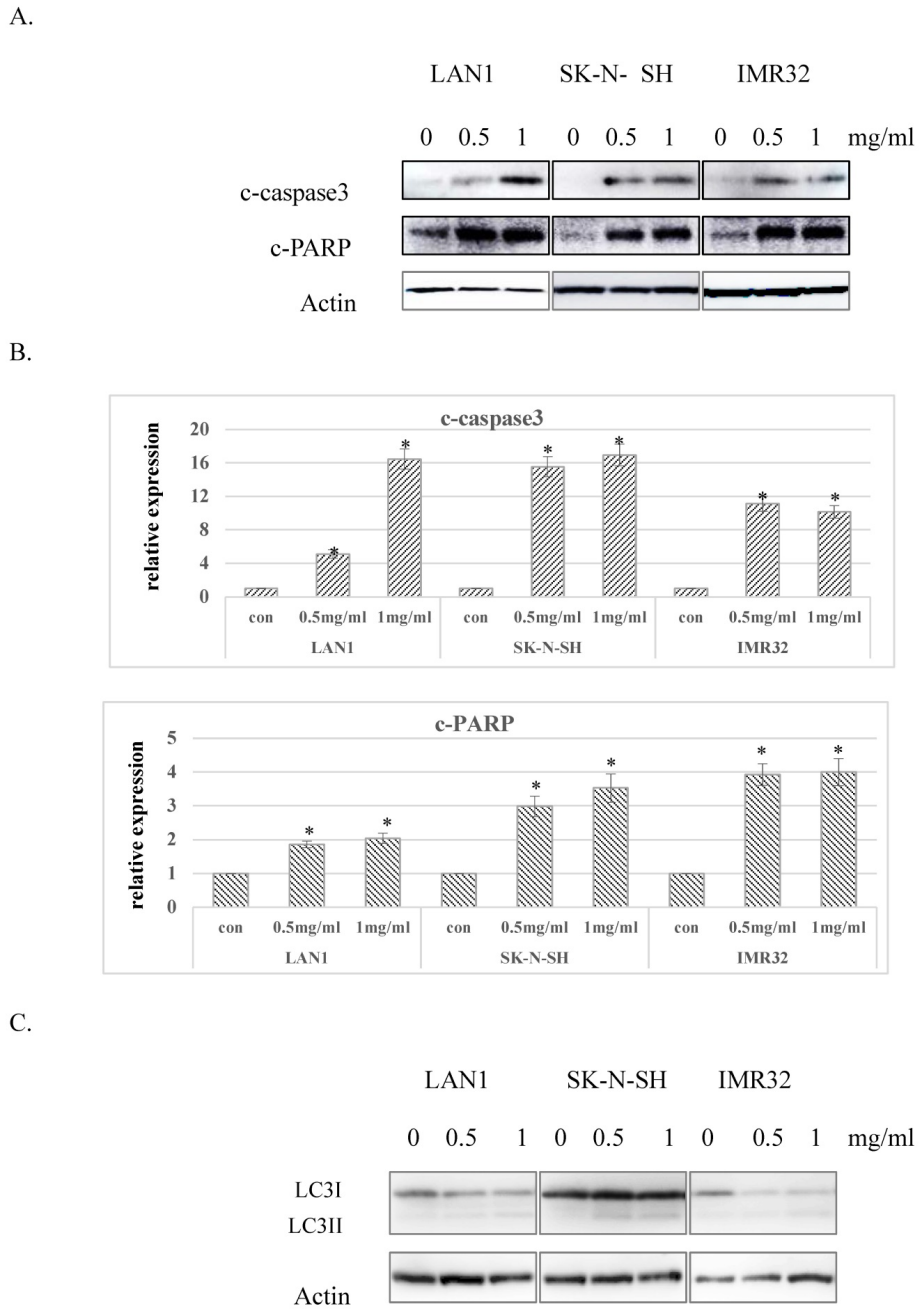
** Effect of Huaier extract on apoptosis and autophagy in neuroblastoma cells.** A. 3 neuroblastoma cell lines were incubated in RPMI1640+10% FBS with/without Huaier extract (0, 0.5, 1 mg/ml) for 48h. Expression of cleaved PARP and cleaved-caspase3 were detected by western blotting as introduced in methods and materials, so was Actin. B. The intensity of cleaved PARP and cleaved-caspase3 were quantified by ImageJ. After normalization to actin, the relative expression of cleaved-PARP and cleaved-caspase3 versus control group was compared. Data represent mean ± SD. * represents P<0.05. C. Effect of Huaier extract on autophagy. 3 neuroblastoma cell lines were incubated in RPMI1640 + 10% FBS with/without Huaier extract (0, 0.5, 1 mg/ml) for 24h. Expression of LC3II and LC3I were detected by western blotting as introduced in materials and methods, so was Actin.

Considering the G0 phase accumulation induced by Huaier extract in the 3 NB cell lines, we further examined whether Huaier extract may induce apoptosis in NB cells through analyzing the expression of apoptosis related proteins using western blotting. After treatment with different doses of Huaier extract for 48 h, as shown in Fig. 3A and Fig. 3B, expression of cleaved-PARP and cleaved-caspase3 were significantly upregulated in all 3 NB cell lines treated with Huaier extract compared to the control cells, demonstrating that Huaier extract induced apoptosis in NB cells. Autophagy is also referred to as programmed cell death type II, as opposed to apoptosis or programmed cell death type I. The conversion of LC3-I to LC3-II has been used as an indicator of autophagy 6. As shown in Fig. 3C, treatment with Huaier extract enhanced the protein ratio of LC3-II/I, implying the activation of autophagy in NB cell lines.

### Huaier extract suppressed MEK/ERK signaling in neuroblastoma cells*.*

**Fig 4:**
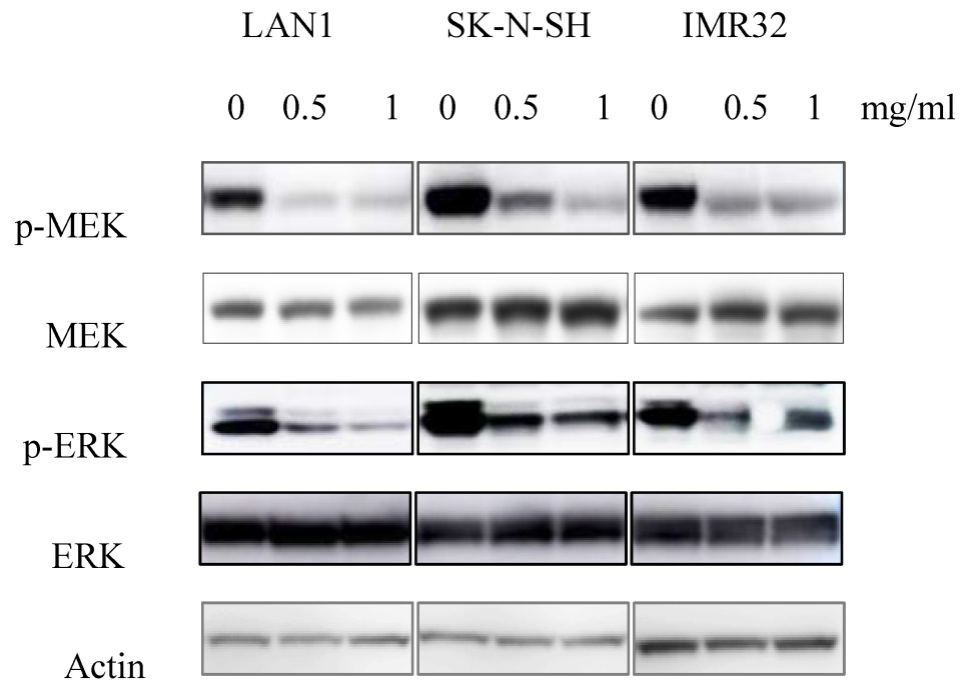
** Effect of Huaier extract on MEK/ERK signaling pathways.** 3 neuroblastoma cell lines were incubated in RPMI1640 + 10% FBS with/without Huaier extract (0, 0.5, 1 mg/ml) for 24h. Total MEK, p-MEK, total ERK and p-ERK were detected by western blotting as introduced in materials and methods, so was Actin.

Since the anti-tumor effect is correlated with down-regulation of cell survival signaling pathways, we hypothesized that Huaier extract may result in the inhibition of the signaling pathways involved in cell survival. MEK has been shown to be involved in the induction of cell cycle related cyclin Ds in many types of cancer 16. Given that treatment with Huaier extract downregulated the expression of cyclinD3 (Fig. 2B), which is one of the downstream factors of ERK^17^, we attempted to determine the changes of MEK/ERK related protein expression after Huaier extract treatment in NB cells. By treating the 3 NB cell lines with different concentrations of Huaier extract, the phosphorylation of MEK and ERK was significantly inhibited in dose-dependent manner (Fig. 4). This finding indicates that Huaier extract may downregulate the expression of cyclinD3 by suppressing the MEK-ERK signaling pathway.

### Huaier extract suppressed mTOR signaling pathway simultaneously in neuroblastoma cells

**Fig 5:**
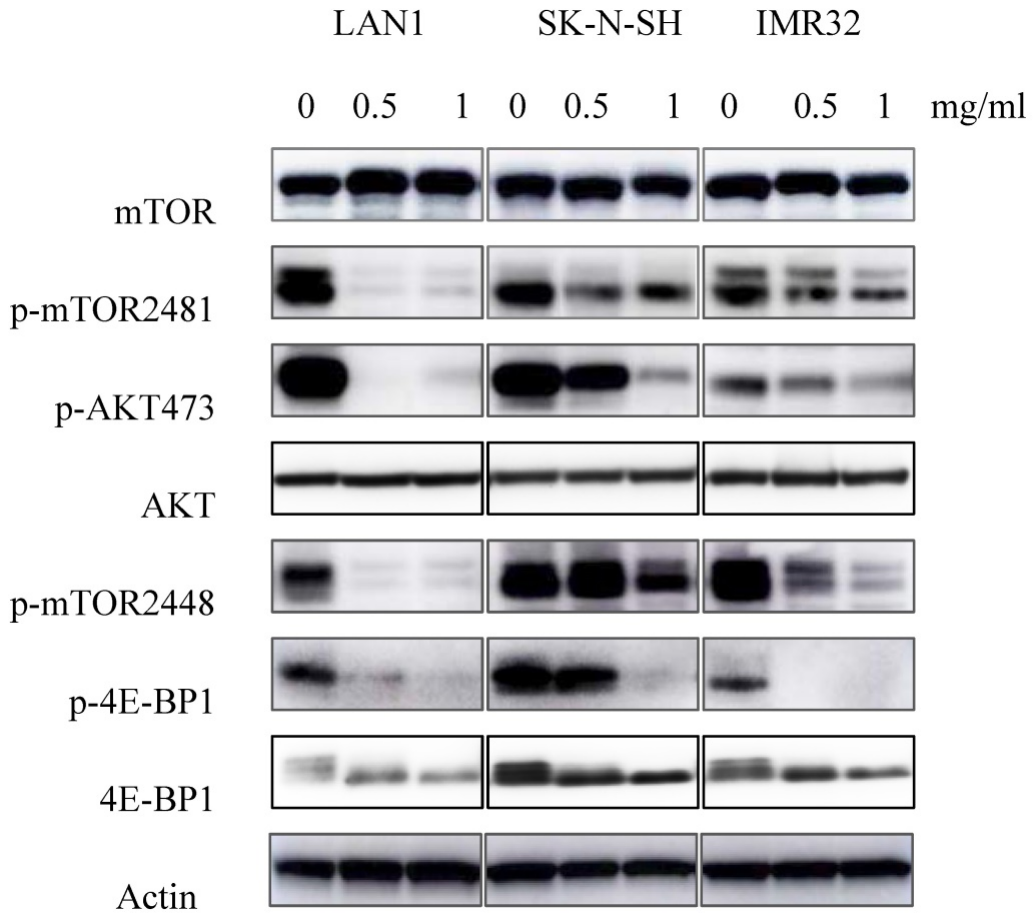
** Effect of Huaier extract on mTOR signaling pathway.** 3 neuroblastoma cell lines were incubated in RPMI1640 + 10% FBS with/without Huaier extract (0, 0.5, 1 mg/ml) for 24h. p-mTOR2481, p-mTOR2448, mTOR, p-AKT 473, AKT, p-4E-BP1, 4E-BP1 were detected by western blotting as introduced in materials and methods, so was Actin.

Previous studies showed the existence of a relationship between the mTOR pathway and NB cell autophagy 6. To determine whether Huaier extract may influence the activity of this pathway, expression of the mTOR signaling cascades was examined using western blotting. As shown in the Fig. 5, Huaier extract significantly inhibited both mTOR S2448 and mTOR S2481 phosphorylation in a concentration dependent manner in the 3 NB cell lines. Furthermore, 4E-BP1 S65 phosphorylation which is downstream targets of mTORC1 and the direct mTORC2 substrate Akt S473 were also efficiently inhibited after Huaier extract treatment in NB cells. These results implied that Huaier extract suppressed activation of mTOR and its downstream molecules in NB cells.

## Discussion

NB is an embryonic solid tumor with poor prognosis for children especially in high-risk group accounting for more than 50% of all NB 18. Generally, therapeutic resistance induced during the clinical courses is one of the major obstacles in patients with advanced-stage, high-risk NB 5. Numerous studies have found aberrant activation of multiple signaling in NB and corresponding targeted therapy also showed promising efficacy. Although promising, mono-target therapy tends to acquire resistance and may preserve tumor cell growth due to activation of other parallel pathways 9,19. Therefore, therapies that influence on multiple tumors promoting survival pathways may be more effective.

With the finding of role in cancer prevention and treatment, TCM has been one of important alternatives among the new developing anti-tumor drugs possessing low toxicity, side effects, low cost^10^. Huaier extract has been evidenced with anti-tumor effect in numerous in pre-clinical studies. It has been used as adjuvant therapy in the clinical treatment courses of cancers such as liver cancer, lung cancer, breast cancer, and gastrointestinal cancer and has exerted a positive effect 15,20. Huaier Extract could inhibit Cell proliferation and in-vivo tumor growth in lung cancer through activating NLRP3-dependent pyroptosis 21.

Our previous study also showed that Huaier extract possesses the potential anti-tumor efficacy in hepatoblastoma cells (data not shown). Furthermore, one multicenter study proved the clinical benefits of Huaier extract which significantly improved recurrence-free survival and reduced extrahepatic recurrence for hepatocellular carcinoma after curative liver resection 15. In the present study, the antitumor effect and underlying mechanisms of Huaier extract in NB cells was investigated.

In the present study, 3 human NB cell lines were incubated with gradient doses of Huaier extract, at a concentration of 0, 0.5, 1, 2, 4, 8 mg/ml. The effects of Huaier extract on the cell-death induction of NB cells were observed. Our results demonstrated that Huaier extract was able to inhibit cell viability and induce changes in cell morphology. MTT assay also revealed that Huaier extract curbed the viability of NB cells in a dose‑dependent manner. In addition, certain apoptotic morphological alteration was observed with Huaier extract treatment at the concentration of 1mg/ml for 72 h, which indicated cell damage.

Previous studies have indicated that Huaier extract also resulted in cell cycle arrest and apoptosis in a number of cell types, such as melanoma, ovarian cancer and breast cancer cells 22-25. Our results revealed that the proportion of G0/G1 phase of all 3 NB cells gradually accumulated, consistent with the role in regulation of the cell-cycle proteins, cyclinD3, was downregulated with an increasing dose of Huaier extract. These results suggest that Huaier extract suppresses the proliferation of NB cells through downregulating cell cycle proteins to induce cell cycle arrest at the G0/G1 phase in a dose‑dependent manner. Induction of the cell death is an expectable treatment method in cancer therapy. In the present study, we analyzed apoptosis induction using western blotting and the results revealed that Huaier extract clearly induced cell apoptosis evidenced by the increasing expression of cleaved-caspase3 and cleaved-PARP in NB cells.

Autophagy can be induced by conditions of cellular stress such as nutrient deprivation, hypoxia, and cytotoxic stress. The mTOR signaling pathway plays a crucial role in modulating autophagy, cell growth, metabolism, survival, and angiogenesis 26. mTOR, a serine/threonine kinase, constitutes the catalytic subunit of two distinct complexes known as mTOR complex 1 (mTORC1) and mTORC2. These two complexes are distinguished by their accessory proteins and their differential sensitivity to rapamycin, as well as by their unique substrates and functions. mTORC1 suppresses catabolic autophagy to prevent a futile cycle in which newly synthesized cellular building blocks are prematurely broken down again^27^. Our previous study proved the overactivation of mTOR signaling in NB cells. In the present study, consisting with the promotion of autophagy by Huaier extract, our results provided its suppress evidence on mTOR signaling in all 3 NB cell lines with the significant decreasing expression of phosphorylation of mTOR and downstream molecules.

Many human tumors and derived cell lines display constitutive activation of MEK/ERK signaling pathway, which regulates cell cycle progression and cell apoptosis 28. Furthermore, our previous study indicated that overactivation of MEK/ERK signaling confers acquired resistance induced by mTOR inhibition in NB cells 9. As shown in present study, the phosphorylation of MEK and ERK in the 3 NB cell lines was suppressed by Huaier extract. That means Huaier extract may suppress the mTOR and MEK/ERK signaling pathways simultaneously in NB.

In summary, our results showed that Huaier extract could inhibit cell proliferation by curbing the mTOR and MEK/ERK signaling to induce apoptosis, autophagy, and cell-cycle arrest in NB cells. To our knowledge, this is a first report to explore the anticancer activity of Huaier extract in NB. Considering the multiple signaling targeted by Huaier extract, it may play a novel role as a complementary medicine in NB treatment.

## Abbreviations

NB: neuroblastoma
ERK: extracellular signal-regulated kinase
MEK: mitogen-activated ERK kinase
TCM: Traditional Chinese Medicine
mTOR: mammalian target of rapamycin
